# The effects of inulin supplementation on eating behaviours in children and adolescents with obesity: a randomized double-blinded placebo-controlled study

**DOI:** 10.1186/s12986-025-00995-0

**Published:** 2025-08-12

**Authors:** Ekkarit Panichsillaphakit, Chonnikant Visuthranukul, Yuda Chongpison, Natthaya Chuaypen, Tanisa Kwanbunbumpen, Jaraspong Uaariyapanichkul, Sirinuch Chomtho

**Affiliations:** 1 Division of Nutrition, Department of Pediatrics, King Chulalongkorn Memorial Hospital, The Thai Red Cross Society, Bangkok, 10330 Thailand; 2 Center of Excellence in Pediatric Nutrition, Division of Nutrition, Department of Pediatrics, Faculty of Medicine, Chulalongkorn University, Bangkok, 10330 Thailand; 3 Center for Excellence in Biostatistics, Research Affairs, Faculty of Medicine, Chulalongkorn University, Bangkok, 10330 Thailand; 4 Metabolic Diseases in Gut and Urinary System Research Unit (MeDGURU), Department of Biochemistry, Faculty of Medicine, Chulalongkorn University, Bangkok, 10330 Thailand

**Keywords:** Children’s eating behaviour questionnaires, Obese children, Satiety, Prebiotic, Inulin

## Abstract

**Background:**

Inulin supplementation may restore gut microbiota dysbiosis and modulate appetite control in childhood obesity. This study evaluated the effects of inulin on eating behaviours and explored their relationships with dietary intake, clinical parameters, and gut microbiota in children with obesity.

**Methods:**

Children aged 7–15 years with obesity were randomly assigned to one of three groups: inulin extracted from Thai Jerusalem artichoke (intervention), maltodextrin (placebo), or dietary fiber advice. All participants received monthly follow-ups with standard dietary and lifestyle guidance for six months. Eating behaviours were assessed at month 0, 3, and 6 using Children’s Eating Behaviour Questionnaires (CEBQs), and their associations with dietary intake, clinical parameters, and gut microbiota were analysed.

**Results:**

A total of 156 children (mean age: 10.4 ± 2.2 years, mean BMI z-score: 3.2 ± 1.0, 58.3% male) completed the study. Emotional undereating (EUE) significantly decreased in the inulin group compared to the placebo group (*p* = 0.01). All groups showed reduced food approach subscales, except emotional overeating (EOE), with no significant differences between groups. Among the food approach subscales, food responsiveness was positively correlated with total calorie and fat intakes at baseline and month 3. EOE showed negative correlation with dietary fiber intake/1,000 kcal at month 6. For the food avoidant subscales, satiety responsiveness negatively related to body weight at baseline and proportion of carbohydrate intake (pCHO) at month 6. Slowness in eating was negatively correlated with BMI z-score and pCHO at the end of the study. A 1-point increase in the desire to drink Likert score was associated with a 62.5 mg/day increase in cholesterol intake post-intervention (95%CI: 16.6-108.4). Glucagon like peptide-1 (GLP-1) was inversely correlated with EOE after the intervention. For every 50 ng/L increase in GLP-1, EOE increased by 0.007 points pre-intervention and decreased by 0.037 points post-intervention. CEBQs showed significant associations with *Agathobacter* at baseline, and with *Oscillibacter*, *UBA1819*, and *Lachnospiraceae_NK4A136* at month 3.

**Conclusions:**

Inulin supplementation influenced eating behaviours, particularly reducing EUE. Significant associations between subjective eating behaviours, dietary intake, biochemical markers, and gut microbiota were observed. These findings suggest that inulin supplementation may be a potential strategy for managing childhood obesity through appetite modulation and improving eating behaviours.

**Trial registration:**

Retrospectively registered at ClinicalTrials.gov (NCT03968003). Registered 30 May 2019.

**Supplementary Information:**

The online version contains supplementary material available at 10.1186/s12986-025-00995-0.

## Introduction

Childhood obesity has dramatically increased over recent decades, posing a significant threat to global public health. The World Health Organization (WHO) Global Health Observatory reported that, in 2017 and 2020, over 340 million children and adolescents aged 5–19 years and nearly 39 million children under the age of five were living with overweight or obesity [1]. Currently, the Southeast Asian Nutrition Surveys II had been reported that the prevalence of overweight and obesity in Thai children was approximately 30% of population in 2019 [2]. Various factors contribute to childhood obesity, including genetics, environment, low socioeconomic status, sedentary lifestyle, dietary patterns and eating behaviours [3]. In general, the obesity is associated with several comorbidities, including hypertension, dyslipidemia, type 2 diabetes, and psychiatric disorders such as anxiety, depression, or eating disorders, even in children affected by this condition [3, 4]. Obesity management typically involves three standard approaches: reducing caloric intake through dietary changes, increasing daily physical activity, and implementing intensive cognitive behavioural therapy [3]. However, sustaining these interventions to achieve long-term success remains a significant challenge.

Children with obesity often experience psychiatric issues that affect their eating behaviours and make them highly sensitive to external food cues [5, 6]. Parental skills, dietary patterns, and food preferences in early childhood significantly shape eating behaviours that persist into adulthood [7]. Studies by Santos et al. [8] and Malczyk et al. [9] found childhood obesity positively correlated with food approach behaviours and inversely associated with food avoidance. Faster eating speed is a hallmark of obesity risk in these children [5]. Moreover, consumptive responses to stress or emotional eating—defined as eating in response to emotional distress, either overeating or undereating rather than hunger—may lead individuals living with obesity to consume imbalanced energy and nutrient intakes, contributing to obesity [10]. Therefore, early management of obesity is essential for establishing healthy eating habits in this population.

Inulin-type fructans (ITFs) have been extensively studied as prebiotics in the management of obesity [11–13]. ITFs are fermented by colonic bacteria in the distal ileum and proximal colon, producing postbiotic metabolites known as short chain fatty acids (SCFAs), which stimulate the secretion of gut-derived hormones from intestinal L-cells. These hormones can regulate appetite through gut-brain signalling pathways [14]. Another plausible underlying mechanism involves inulin-derived SCFAs stimulating the release of glucagon like peptide-1 (GLP-1) and peptide YY (PYY), which slow intestinal transit and delay gastric emptying [15]. Consequently, ITFs may reduce energy intake, enhance satiety, and improve body composition [11, 16]. Previous studies have shown that ITFs consumption in adults with overweight or obesity could modulate satiation, reduce hunger, and decrease the desire to eat [17–19]. In children, evidence is limited to one clinical trial, Hume et al. [20] reported that inulin supplementation increased fullness and reduced energy intakes after 16 weeks in the experimental group compared to placebo. This highlights the scarcity of clinical studies examining the effects of inulin on satiety regulation in children with obesity. Therefore, this study aimed to evaluate the effects of inulin supplementation on subjective eating behaviours and explore their relationships with dietary intake, clinical parameters, and gut microbiota in children with obesity.

## Methods

### Participants

This study was a three-arm randomized placebo-controlled trial conducted from August 2017 to July 2020 at the King Chulalongkorn Memorial Hospital, Bangkok, Thailand, with detailed methodology was published elsewhere [12]. The study protocol was approved by the Institutional Review Board of the Faculty of Medicine, Chulalongkorn University (IRB no. 240/60). Written informed consent was obtained from all participants and their guardians, and children aged 7 to 15 years provided assent by signing assent forms. This trial was registered at ClinicalTrials.gov (NCT03968003) and reported in accordance with the CONSORT statement for randomized trials. Children and adolescents aged 7-15 years who met the WHO criteria for obesity [body mass index (BMI) > median plus 2 standard deviations (SDs)] were recruited, as described in the previous study [12].

### Study design

Briefly, 165 participants were randomly assigned to one of the three groups: inulin (intervention), placebo, and dietary fiber advice. However, nine participants (5%) dropped out throughout the study due to personal reasons (e.g. travel inconvenience). Participants and investigators involved in the intervention and placebo groups were blinded to group allocation to preserve the double-blind design. The intervention group consumed 13 g of inulin extracted from Thai Jerusalem artichoke by our patent technique (Patent no. 15858) administered once daily before dinner. The placebo group received 11 g of isocaloric maltodextrin, while the dietary fiber advice group received guidance based on age-appropriate intake recommendations [21, 22]. All groups were provided with the same dietary guidance on energy intake, daily physical activity, and lifestyle modifications. Participants were followed monthly, and the Children’s Eating Behaviour Questionnaires (CEBQs) were administered at baseline, month 3, and month 6. Additional details and the study flow diagram have been published previously [12].

### Dietary assessment

The three-day dietary intakes (two weekdays and one weekend day) were reported at baseline, month 3 and month 6 by the children’s parents/guardians, who were provided with instructions on how to complete the record form. Subsequently, a proficient dietitian reviewed the accuracy of the recorded food data and calculated the daily energy intake, percentage of caloric distributions-defined as the ratio of calories from each macronutrient to the total energy intake, nutrient and fiber intakes using the Institute of Nutrition, Mahidol University Calculation-Nutrients (INMUCALs) Version 3 [23].

### Anthropometric measurement

Trained personnel conducted anthropometric measurement, as detailed previously [12]. In brief, weight and height were measured without shoes and with light clothing using a stadiometer to the nearest 0.1 kg and to the nearest 0.1 cm, respectively. Waist circumference was assessed at the umbilical level after normal exhalation in standing position. BMI was calculated as weight in kilograms divided by the square of height in meters (kg/m^2^), and BMI z-score was calculated based on the WHO 2007 growth reference using the WHO Anthroplus program Version 1.0.0 [24].

### Eating behaviour questionnaires

The development and validation of the CEBQs have been reported previously [25, 26]; hence, further validation was not necessary in the context of our present study. In brief, the CEBQs consist of eight subscales with 35 items, equally divided into four food approach subscales, including: 1) food responsiveness (FR), which reflects a child’s response to environmental food cues (e.g., “Given the choice, my child would eat most of the time”); 2) enjoyment of food (EF), which represents the desire to eat and the pleasure derived from eating (e.g., “My child is interested in food”); 3) emotional overeating (EOE), which reflects increased food consumption in response to negative emotions (e.g., “My child eats more when annoyed”); and 4) desire to drink (DD), which reflects a tendency to consume sweetened beverages (e.g., "If given the chance, my child would drink continuously throughout the day”). On the other hand, the four food avoidance subscales include: 5) satiety responsiveness (SR), which reflects reduced hunger following food intake (e.g., “My child gets full up easily”); 6) slowness in eating (SE), which represents a decreased speed of eating over the course of meal (e.g., “My child finishes his/her meal quickly”); 7) emotional undereating (EUE), which reflects decreased food intake in response to negative emotions (e.g., “My child eats less when angry”); and 8) food fussiness (FF), which represents picky eating or limited intake of unfamiliar foods (e.g., “My child is interested in tasting food s/he hasn’t tasted before”). Each subscale comprises 3-6 items rated on a five-point Likert scale. For children aged 7-12 years, the questionnaire was completed by their parents/guardians, while adolescents aged 12 and older self-reported their responses. Additionally, experts in child development were consulted in person to evaluate the clarity and wording of the questionnaires. Based on their feedback, revisions were made to improve response rates and reduce respondent burden.

### Gut hormone analysis

Following randomization, with estimated difference in GLP-1 hormone level of 5.7 and an SD of 6.61 based on a power of 0.8 to detect a significant difference (*p* = 0.05, 2-sided), a minimum of 66 participants (*n* = 22/group) were required [20]; 5 additional participants/group were added to account for 20% dropouts. Therefore, 85 participants with available 24-hour fasting blood samples pre- and post-intervention were analysed for GLP-1 and PYY hormones, using Human Glucagon-like Peptide 1 and Human Peptide YY ELISA Kits (MyBioSource, Inc., USA). Afterward, blood was processed within 30 min, and serum samples were stored at -80 °C until analysis. Both hormones were quantified using the Sandwich ELISA technique, with detection ranges of 15-3000 ng/L (sensitivity: 7.29 ng/L) for GLP-1 and 3-900 pg/mL (sensitivity: 1.56 pg/mL) for PYY.

### Gut microbiota analysis

Fresh fecal samples were collected at baseline, month 3, and month 6 for gut microbiota analysis, as detailed previously [27]. Participants used sterile stool collection kits and stored samples in a household freezer before delivering them to the lab within 24 h, where they were stored at -80 °C until analysis. DNA was extracted using the QIAamp Fast DNA Stool Mini Kit (QIAGEN, Germany) following the manufacturer’s instructions. For 16 S rRNA sequencing, Illumina paired-end reads from all-time points were analysed using QIIME2 (v.2020.8) [28]. Quality was inspected with FastQC [29], and operational taxonomic units were defined at a 97% similarity threshold. Chimeric reads were removed with q2-dada2 [30]. Taxonomic annotation used a Naïve Bayes classifier trained on the SILVA 16 S rRNA database (v.132) [31, 32], with unclassified sequences labeled as ‘unknown’. Linear Discriminant Analysis Effect Size: LEfSe [cut-off linear discriminant analysis (LDA) score ≥ 2.0, *p*-value < 0.05] were subsequently performed to identify the taxonomic differences among baseline, month 3, and month 6 groups [33].

### Statistical analysis

The normality of the data was evaluated using the Shapiro-Wilk test. Descriptive characteristics were presented as frequency (%) for categorical variables and mean ± SDs for continuous variables with normal distributions. Comparisons among the three groups were conducted using one-way analysis of variance (ANOVA) for continuous variables and chi-square tests for categorical variables. The random-effects models were used to compare the means of CEBQs at baseline and follow-up periods, considering changes both within- and between-groups over time. Linear mixed-effect models were used to evaluate the effects of intervention on CEBQs over 6 months and the impacts of variables over time, with interactions tested between intervention groups and time, and between CEBQs and time. An interaction *p*-value < 0.15 was considered significant. Linear regression analysis was used to develop a six-month prediction model of the accumulative subjective eating behaviour data at month 0, 3, 6 with changes in dietary intakes and biochemical markers over the six-month period (difference of month 6 minus month 0). Additionally, CEBQs from months 0 and 6 were examined as averages to identify predictions of dietary intakes and biochemical markers at baseline and month 6, using linear mixed-effect models. Spearman correlation coefficients were used to determine associations between CEBQs and gut microbiota at baseline, month 3, and month 6 of the study.

All statistical tests were two-sided, and a *p*-value < 0.05 was considered statistically significant. Analyses were performed using STATA version 18.0 (STATA Statistical Software: Release 18. College Station, TX, USA).

## Results

A total of 156 randomized children (*n* = 114) and adolescents (*n* = 42) with obesity (mean age: 10.4 ± 2.2 years, mean BMI z-score: 3.2 ± 1.0, 58.3% male) completed the study, flow diagram was shown in **Appendix 1**. Demographic and baseline characteristics are presented in Table 1. No significant differences were observed in baseline anthropometric measures and nutrient intakes among the three groups. The eating behaviour subscales showed no significant variation across the three groups at baseline. Overall, the four food approach subscales had mean scores higher than the food avoidance subscales, except for EUE and FF, which exceeded the mean score of the EOE subscale. Among the food approach subscales, EF had the highest mean score, while FF, reflecting picky eating, had the highest mean score among the food avoidance subscales.

**Table 1:** Demographic data, nutrient intakes, clinical parameters, and eating behaviour assessments of the participants at baseline

	Placebo (*n* = 52)	Inulin (*n* = 52)	Dietary fiber advice (*n* = 52)	*p*-value
Age, years	10.7 ± 2.5	10.3 ± 2.2	10.3 ± 2.0	0.52
Sex, male, %	53.8	55.8	65.4	
BMI, kg/m^2^	28.5 ± 4.7	28.4 ± 4.6	27.5 ± 3.4	0.46
BMI z-score	3.2 ± 1.1	3.3 ± 1.0	3.2 ± 1.0	0.84
Waist circumference, cm	90.6 ± 11.1	90.0 ± 11.5	88.5 ± 10.2	0.62
**Nutrient intakes**				
Total caloric intake, kcal/d	1416.4 ± 542.1	1435.8 ± 557.1	1433.9 ± 472.1	0.98
Total protein intake, g/kg/d	1.0 ± 0.4	1.0 ± 0.4	1.1 ± 0.1^§^	0.46
Total fat intake, g/d	56.6 ± 28.4	59.3 ± 30.0	57.2 ± 27.3	0.88
Cholesterol intake, mg/d	306.6 ± 27.8^§^	324.7 ± 35.1^§^	328.5 ± 32.4^§^	0.88
Dietary fiber intake, g/1,000 kcal	2.9 ± 0.3^§^	2.8 ± 0.3^§^	2.6 ± 0.3^§^	0.79
Caloric distribution, % C : P : F	48 : 16 : 36	48 : 16 : 36	48 : 16 : 36	
**Gut hormones** ^**†**^				
GLP-1, ng/L	258.5 ± 30.8^§^	276.1 ± 41.4^§^	347.5 ± 69.4^§^	0.40
PYY, pg/mL	82.7 ± 7.7^§^	88.0 ± 9.6^§^	99.5 ± 13.1^§^	0.69
**CEBQs**				
Food responsiveness (FR)	3.2 ± 1.0	3.1 ± 0.9	3.3 ± 0.9	0.45
Enjoyment of food (EF)	3.9 ± 0.8	4.0 ± 0.7	4.0 ± 0.7	0.56
Emotional overeating (EOE)	2.3 ± 1.0	2.0 ± 0.8	2.1 ± 0.8	0.25
Desire to drink (DD)	3.2 ± 1.3	3.1 ± 1.1	3.1 ± 1.2	0.82
Satiety responsiveness (SR)	2.2 ± 0.6	2.1 ± 0.5	2.1 ± 0.5	0.50
Slowness in eating (SE)	2.0 ± 0.8	2.1 ± 0.8	2.0 ± 0.8	0.56
Emotional undereating (EUE)	2.6 ± 0.8	2.2 ± 0.6	2.3 ± 0.8	0.06
Food fussiness (FF)	2.7 ± 0.9	2.8 ± 0.9	2.7 ± 0.8	0.62

The data were presented as percentage and means ± SD or SE. Chi-Square test of independence and one-way ANOVA were used to compare categorical and continuous variables, respectively. ^†^Satiety hormones were available from 85 participants (30 placebo group, 29 inulin group, and 26 dietary fiber advice group). CEBQs were assessed using 5-point Likert scales.

Abbreviations: *BMI*, body mass index; *CEBQs*, Children’s Eating Behaviour Questionnaires; *C*, carbohydrate; *P*, protein; *F*, fat; *GLP-1*, glucagon-like peptide 1; *PYY*, peptide YY

### Changes in subjective eating behaviours over the 6-month study

**Fig. 1:**
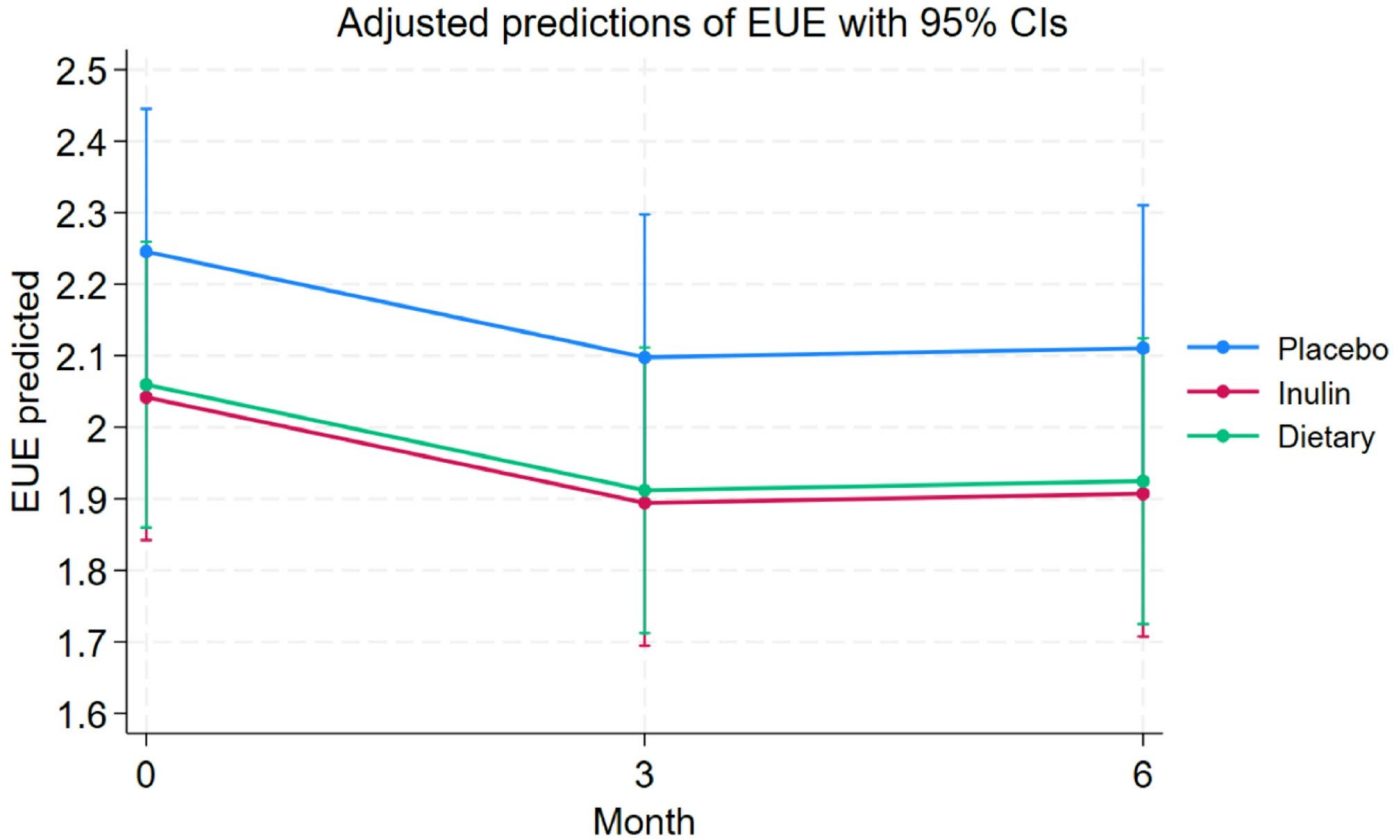
Changes in EUE subscale over 6 months among the three groups of children with obesity. EUE significantly decreased in the inulin group (effect size − 0.29 [95% CI -0.51, -0.06], *p* = 0.01) and dietary fiber advice group (effect size − 0.24 [95% CI -0.46, -0.01], *p* = 0.04) compared to the placebo group, analysed by linear mixed-effect models. Abbreviations: *EUE*, emotional undereating

After completing the study, all groups of children and adolescents with obesity demonstrated a significant reduction in BMI z-scores (*p* < 0.05). The effects of the intervention groups on eating behaviours over 6 months were assessed using linear mixed-effect models. Significant changes were observed in the EUE subscale, with greater reductions in the inulin group compared to placebo (*p* = 0.01) and in the dietary fiber advice group compared to placebo (*p* = 0.04) (Fig. 1). Additionally, all food approach subscales, except EOE, showed significant and sustained decreases from baseline to 3 and 6 months across all three groups (*p* < 0.001).

### Associations between eating behaviours, dietary intake, and clinical parameters at baseline, month 3, and month 6

The associations between eating behaviour scores and various variables were analysed at baseline, month 3, and month 6. At baseline, for food approach, FR was positively correlated with total calorie (TC) and fat intakes (FATi) (*r* = 0.23, *p* = 0.005 and *r* = 0.25, *p* = 0.003, respectively), whereas EF subscale was positively correlated with cholesterol intake (*r* = 0.18, *p* = 0.03). In contrast, SR was negatively correlated with body weight and FF was negatively associated with body weight for height (*r* = -0.24, *p* = 0.004 and *r* = -0.18, *p* = 0.03, respectively). At month 3, FR continued to show a significant positive correlation with TC and FATi (*r* = 0.28, *p* = 0.001 and *r* = 0.21, *p* = 0.008, respectively). EF was positively correlated with BMI z-score and TC (*r* = 0.20, *p* = 0.01 and *r* = 0.17, *p* = 0.04, respectively). EOE subscale was positively associated with TC, FATi, and proportion of fat intake (*r* = 0.26, *p* = 0.001, *r* = 0.29, *p* < 0.0001, and *r* = 0.22, *p* = 0.007, respectively), whereas it was negatively correlated with dietary fiber intake per 1,000 kcal (DF/Kcal) (*r* = -0.18, *p* = 0.02). By month 6, among the food approach subscales, EOE consistently showed a significantly negative correlation with DF/Kcal (*r* = -0.19, *p* = 0.02). For the food avoidant subscales, SR was negatively associated with proportion of carbohydrate intake (pCHO) (*r* = -0.24, *p* = 0.003), and SE was negatively correlated with BMI z-score and pCHO (*r* = -0.18, *p* = 0.03 and *r* = -0.17, *p* = 0.04, respectively), as determined by Spearman correlation coefficients (Table 2).

**Table 2:** Associations of CEBQ subscales with dietary intake and clinical parameters at baseline, 3 months, and 6 months among participants who completed the intervention (*n* = 156)

Factors	Baseline						Month 3						Month 6					
	Children eating behaviour questionnaires (95% CI)						Children eating behaviour questionnaires (95% CI)						Children eating behaviour questionnaires (95% CI)					
	FR	EF	EOE	SR	SE	FF	FR	EF	EOE	SR	SE	FF	FR	EF	EOE	SR	SE	FF
Body weight	-0.01 (-0.18 to 0.15)	-0.03 (-0.20 to 0.14)	0.06 (-0.11 to 0.23)	-0.24** (-0.39 to -0.07)	-0.14 (-0.30 to 0.03)	-0.14 (-0.30 to 0.03)	-0.04 (-0.20 to 0.12)	0.01 (-0.16 to 0.17)	0.14 (-0.03 to 0.29)	-0.09 (-0.25 to 0.07)	-0.11 (-0.27, 0.06)	-0.05 (-0.21 to 0.12)	0.03 (-0.13 to 0.19)	-0.01 (-0.16 to 0.16)	0.08 (-0.08, to 0.24)	-0.16 (-0.32 to 0.01)	-0.12 (-0.28 to 0.04)	0.08 (-0.08 to 0.24)
Body weight for height	0.01 (-0.16 to 0.17)	-0.02 (-0.19 to 0.15)	0.06 (-0.11 to 0.22)	-0.17 (-0.30 to 0.01)	0.01 (-0.16 to 0.18)	-0.18* (-0.34 to -0.01)	0.05 (-0.11 to 0.21)	0.09 (-0.08 to 0.25)	0.29 (-0.14 to 0.19)	-0.03 (-0.19 to 0.14)	-0.04 (-0.20, 0.12)	-0.12 (-0.27 to 0.05)	0.02 (-0.14 to 0.19)	-0.04 (-0.20 to 0.12)	-0.07 (-0.23 to 0.10)	0.02 (-0.14 to 0.18)	-0.05 (-0.21 to 0.12)	-0.04 (-0.20 to 0.13)
BMI z-score	0.04 (-0.13 to 0.21)	0.08 (-0.09 to 0.24)	0.07 (-0.10 to 0.23)	-0.08 (-0.24 to 0.09)	-0.08 (-0.25 to 0.08)	-0.12 (-0.28 to 0.05)	0.14 (-0.03 to 0.29)	0.20* (0.04 to 0.35)	0.07 (-0.09 to 0.23)	-0.12 (-0.27 to 0.05)	-0.13 (-0.29, 0.04)	-0.05 (-0.21 to 0.11)	0.05 (-0.11 to 0.21)	0.06 (-0.10 to 0.22)	-0.04 (-0.20 to 0.13)	-0.07 (-0.23 to 0.09)	-0.18* (-0.33 to -0.01)	-0.03 (-0.19 to 0.13)
Total calories intake	0.23** (0.07 to 0.39)	0.11 (-0.06 to 0.27)	0.11 (-0.05 to 0.28)	-0.16 (-0.32 to 0.01)	-0.08 (-0.25 to 0.09)	0.01 (-0.16 to 0.18)	0.28** (0.12 to 0.42)	0.17* (0.01 to 0.32)	0.26** (0.10 to 0.41)	-0.12 (-0.28 to 0.04)	-0.03 (-0.19, 0.14)	-0.06 (-0.22 to 0.11)	0.09 (-0.07 to 0.25)	0.09 (-0.07, 0.25)	0.05 (-0.12 to 0.21)	-0.06 (-0.22 to 0.10)	-0.08 (-0.24 to 0.08)	-0.03 (-0.19 to 0.14)
Dietary fiber intake	0.02 (-0.15 to 0.19)	0.06 (-0.10 to 0.23)	-0.03 (-0.20 to 0.14)	-0.06 (-0.22 to 0.11)	-0.14 (-0.30 to 0.03)	-0.12 (-0.28 to 0.05)	-0.05 (-0.21 to 0.12)	0.07 (-0.10 to 0.23)	-0.18* (-0.34 to -0.02)	-0.04 (-0.21 to0.12)	0.08 (-0.09, 0.24)	-0.11 (-0.27 to 0.05)	0.02 (-0.18 to 0.15)	0.05 (-0.11, 0.21)	-0.19* (-0.34 to -0.03)	-0.26** (-0.40 to -0.10)	-0.08 (-0.24 to 0.08)	-0.08 (-0.24 to 0.09)
Total fat intake	0.25** (0.08 to 0.40)	0.15 (-0.02 to 0.31)	0.12 (-0.05 to 0.28)	-0.11 (-0.27 to 0.06)	0.01 (-0.17 to 0.17)	0.10 (-0.07 to 0.27)	0.22** (0.05 to 0.37)	0.15 (-0.01 to 0.31)	0.29*** (0.14 to 0.44)	-0.02 (-0.18 to 0.15)	-0.07 (-0.24, 0.09)	0.01 (-0.15 to 0.18)	0.02 (-0.15 to 0.18)	0.03 (-0.14, 0.19)	0.08 (-0.09 to 0.24)	0.10 (-0.06 to 0.26)	0.03 (-0.13 to 0.19)	-0.03 (-0.19 to 0.13)
Total cholesterol intake	0.11 (-0.06 to 0.27)	0.18* (0.01 to 0.33)	0.03 (-0.14 to 0.20)	0.02 (-0.15 to 0.18)	0.01 (-0.16 to 0.18)	-0.04 (-0.21 to 0.13)	0.16 (-0.01 to 0.31)	0.10 (-0.06 to 0.26)	0.15 (-0.02 to 0.30)	-0.08 (-0.24 to 0.09)	-0.14 (-0.30, 0.02)	-0.07 (-0.23 to 0.10)	0.03 (-0.13 to 0.19)	0.09 (-0.08, 0.25)	-0.01 (-0.17 to 0.16)	0.06 (-0.11 to 0.22)	0.02 (-0.15 to 0.18)	-0.13 (-0.29 to 0.04)
Proportion of carbohydrate intake	-0.11 (-0.28 to 0.05)	-0.15 (-0.31 to 0.01)	-0.07 (-0.24 to 0.10)	0.03 (-0.14 to 0.19)	0.01 (-0.16 to 0.17)	-0.04 (-0.21 to 0.13)	-0.03 (-0.19 to 0.14)	-0.02 (-0.19 to 0.14)	-0.22** (-0.37 to -0.06)	-0.11 (-0.27 to 0.05)	0.02 (-0.15, 0.18)	-0.08 (-0.24 to 0.09)	0.13 (-0.04 to 0.28)	0.06 (-0.10, 0.23)	-0.10 (-0.26 to 0.07)	-0.24** (-0.39 to -0.08)	-0.17* (-0.32 to -0.01)	0.01 (-0.15 to 0.18)
Proportion of protein intake	-0.10 (-0.26 to 0.07)	0.03 (-0.14 to 0.20)	-0.01 (-0.17 to 0.16)	-0.04 (-0.20 to 0.13)	-0.06 (-0.22 to 0.11)	-0.06 (-0.23 to 0,13)	0.03 (-0.14 to 0.19)	0.01 (-0.16 to 0.17)	0.01 (-0.16 to 0.17)	-0.05 (-0.21 to 0.12)	0.06 (-0.11, 0.22)	-0.03 (-0.19 to 0.14)	-0.04 (-0.21 to 0.12)	0.03 (-0.13, 0.19)	0.11 (-0.05 to 0.27)	0.10 (-0.07 to 0.25)	0.05 (-0.12 to 0.21)	0.05 (-0.11 to 0.21)
Proportion of fat intake	0.16 (-0.01 to 0.32)	0.14 (-0.03 to 0.30)	0.06 (-0.11 to 0.22)	-0.04 (-0.21 to 0.12)	0.02 (-0.15 to 0.19)	0.09 (-0.08 to 0.26)	0.22 (-0.05 to 0.37)	0.02 (-0.15 to 0.18)	0.22** (0.06 to 0.37)	0.12 (-0.04 to 0.28)	-0.07 (-0.24, 0.09)	0.11 (-0.06 to 0.27)	-0.09 (-0.25 to 0.07)	-0.06 (-0.22, 0.10)	0.05 (-0.11 to 0.21)	0.22** (0.06 to 0.37)	0.12 (-0.04 to 0.28)	-0.03 (-0.19 to 0.13)

Associations were analysed by using Spearman correlation coefficients

*Statistical significance at *p* < 0.05, **Statistical significance at *p* < 0.01, ***Statistical significance at *p* < 0.001

Abbreviations: *CEBQs*, Children’s Eating Behaviour Questionnaires; *BMI*, body mass index; *FR*, food responsiveness; *EF*, enjoyment of food; *EOE*, emotional overeating; *SR*, satiety responsiveness; *SE*, slowness in eating; *FF*, food fussiness

**Fig. 2:**
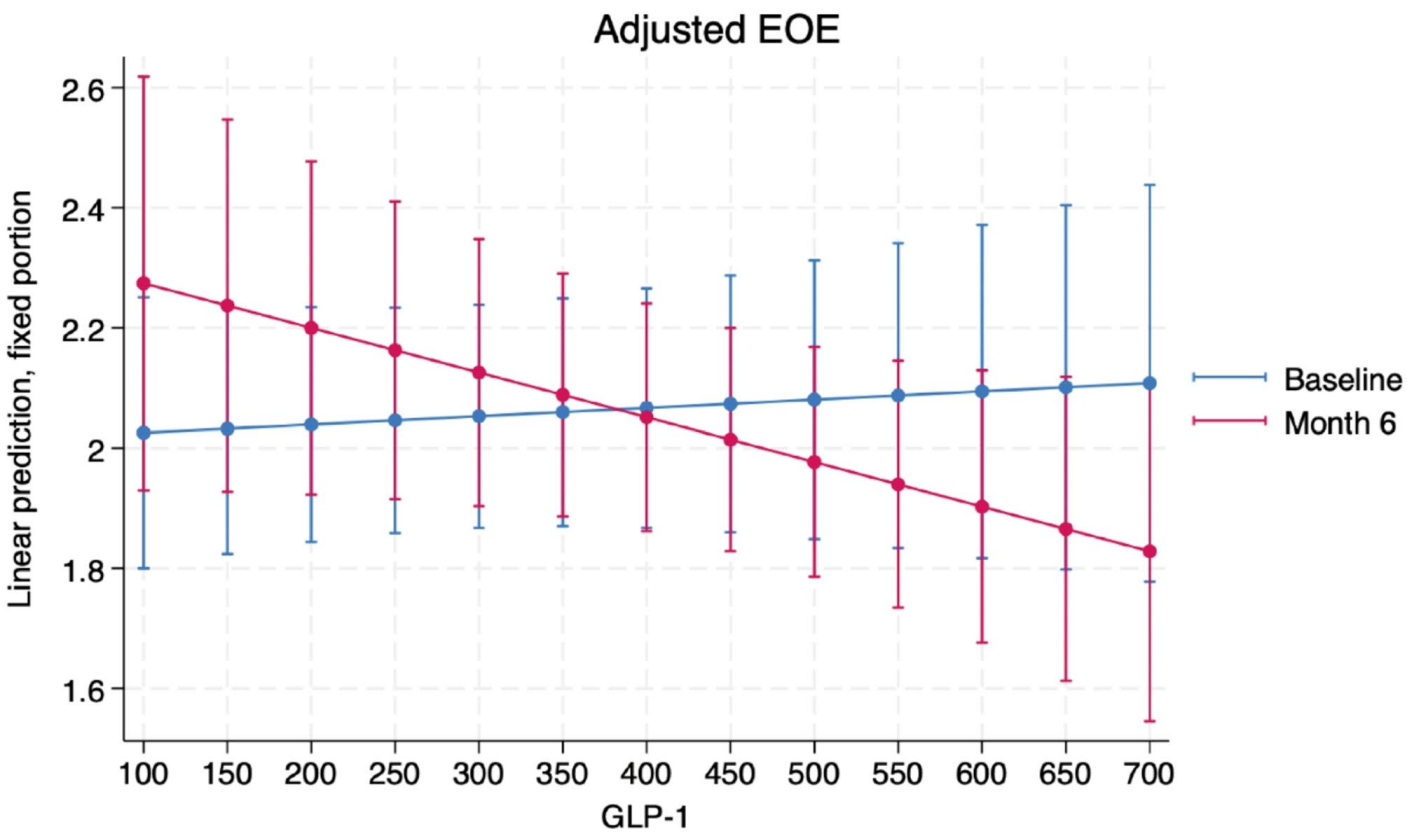
GLP-1 was significantly inversely correlated with the change in the EOE subscale among participants with complete satiety hormone measurements (*n* = 85). The EOE subscale and GLP-1 data at baseline and at month 6 were used for analysis. For every 50 ng/L increase in GLP-1, the EOE subscale increased by 0.007 points pre-intervention and decreased by 0.037 points post-intervention, analysed by linear mixed-effect model. Abbreviations: *EOE*, emotional overeating; *GLP-1* glucagon like peptide-1

Moreover, a six-month predictive model showed that each 1-point increase on the Likert scale of DD was associated with a 62.5 mg/day increase in cholesterol intake (95% CI: 16.58 to 108.35). Regarding satiety-related hormones, which were measured in 85 children and adolescents at baseline and at the final visit, GLP-1 was significantly inversely correlated with the change in the EOE subscale after the intervention. For every 50 ng/L increase in GLP-1, the EOE subscale increased by 0.007 points pre-intervention and decreased by 0.037 points post-intervention (Fig. 2).

### Associations between subjective eating behaviours and gut microbiota at baseline, month 3, and month 6

**Fig. 3:**
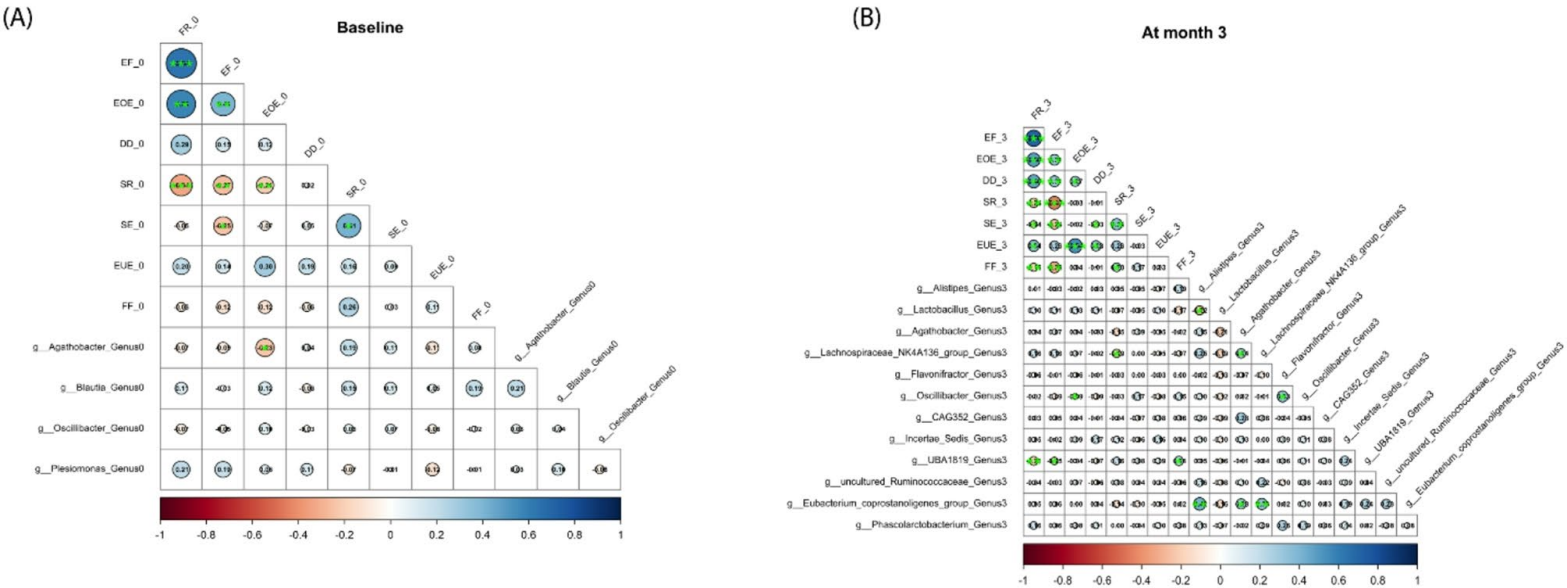
Heatmap of associations between subjective eating behaviors and gut microbiota at baseline and month 3. (**A**) At baseline (*n* = 154), EOE was negatively correlated with *Agathobacter* (*r* = -0.234, *p* = 0.019). (**B**) At month 3 (*n* = 148), EF and FR were negatively associated with *UBA1819* (*r* = -0.15, *p* = 0.019 and *r* = -0.212, *p* = 0.006, respectively). EOE was negatively correlated with *Oscillibacter* (*r* = -0.094, *p* = 0.046). FF positively associated with *UBA1819* (*r* = 0.237, *p* = 0.049), while SR was negatively associated with *Lachnospiraceae_NK4A136* (*r* = -0.19, *p* = 0.042), analysed by Spearman correlation coefficients. *Statistical significance at *p* < 0.05, **Statistical significance at *p* < 0.01, ***Statistical significance at *p* < 0.001. Abbreviations: *DD*, desire to drink; *EF*, enjoyment of food; *EOE*, emotional overeating; *EUE*, emotional undereating; *FF*, food fussiness; *FR*, food responsiveness; *SE*, slowness in eating; *SR*, satiety responsiveness

The relationships between eating behaviour scores and gut microbiota were analysed at baseline, month 3, and month 6. First, bacterial signatures at the genus level among the groups were identified using LEfSe analysis. There were 4, 12, and 4 signature genera at baseline, month 3, and month 6, respectively (Supplementary Fig. 1). Next, the signature bacteria from each group were correlated with eating behaviour scores. The results demonstrated that, at baseline, EOE was negatively correlated with *Agathobacter* (*r* = -0.234, *p* = 0.019) (Fig. 3A). At month 3, for food approach, EF and FR were negatively associated with *UBA1819* (*r* = -0.15, *p* = 0.019 and *r* = -0.212, *p* = 0.006, respectively). EOE was negatively correlated with *Oscillibacter* (*r* = -0.094, *p* = 0.046). For food avoidance, FF was positively associated with *UBA1819* (*r* = 0.237, *p* = 0.049), while SR was negatively associated with *Lachnospiraceae_NK4A136* (*r* = -0.19, *p* = 0.042) (Fig. 3B). There were no associations between CEBQs and gut microbiota at month 6.

## Discussion

To the best of our knowledge, this was the largest randomized controlled trial to explore the effects of inulin on eating behaviours in children with obesity. Emotional Undereating (EUE) significantly decreased in the inulin and dietary fiber advice groups compared to the placebo group. Significant associations were identified between eating behaviours, dietary intake, and clinical parameters. After the intervention, a higher Desire to Drink (DD) Likert scale was linked to increased cholesterol intake and Emotional Overeating (EOE) was inversely correlated with Glucagon-like peptide 1 (GLP-1). Additionally, several food approaches and avoidance subscales showed significant associations with different microbial taxa over time.

It is widely accepted that children with obesity experience an imbalance between energy intake and expenditure, leading to excessive body fat accumulation [34]. Emotional eating behaviours may be linked to dysregulation of satiety response and sensitivity to environmental food stimuli, which might contribute to childhood obesity. Emotional eating was defined as under- or overeating in response to a negative emotional stimulus. Children with obesity were suffered from several emotional problems, including anxiety, depression, irritability, social isolation, and school bullying [4, 9]. Emotional tension can diminish food perception awareness in these children and may also reduce their ability to cope with stress and maladaptive emotional regulation, thereby exacerbating uncontrolled eating habits [10]. The current study observed that inulin supplementation significantly decreased EUE subscale from baseline to the final visit compared to placebo, which was also found in the dietary fiber advice group. EUE can lead to imbalanced eating patterns, including excessive food restriction or nutrient deficiencies. Furthermore, avoiding food in response to emotions may indicate underlying stress or inadequate emotional regulation. In many cases, children may compensate by overeating when their emotional state improves, potentially exacerbating weight gain and worsening obesity. There was research exploring the effect of inulin on psychiatric measurements. Jackson et al. [35] showed that 8 g of Inulin-type fructans (ITFs) supplement for 5 weeks in healthy adults significantly decreased depression and anxiety scores, while improving sleep quality. They also found that ITFs significantly increased healthy gut bacteria which correlated with several mood parameters. For instance, *Bifidobacterium* was strongly associated with alleviating depression, anxiety, and mood disturbances. This is supported by our previous experiment, which showed that inulin supplementation significantly enhanced *Bifidobacterium* and several beneficial microbes in children with obesity [36]. In addition, it is recognized that dietary fiber improves gut health and promotes feeling of fullness, which may stabilize appetite and eating behaviours [11]. The proposed mechanism underlying these advantages is that intestinal metabolites, produced by gut microorganisms utilizing prebiotics, act as precursors for the synthesizing neurotransmitters, including serotonin, dopamine, and norepinephrine in the human body [37]. These neurohormonal substances regulate mood expression in different brain regions. Therefore, the reduction in EUE observed with inulin supplementation and encouraging a fiber-rich diet can be considered beneficial, as they may help foster balanced eating habits that are less influenced by emotional triggers, whether under- or overeating.

Food preferences and dietary patterns play a crucial role in pediatric eating behaviours. At baseline, our results showed that Food Fussiness (FF) had the highest score in food avoidance, often linked to picky eating and the rejection of unfamiliar foods [38]. While FF is more common in children with lower body mass, children living with obesity may compensate by consuming more energy-dense foods and avoiding healthier options [39]. Hayes et al. [40] found that a reduction in FF correlated with a greater decrease in BMI z-score. Thus, decreasing FF may improve dietary diversity and reduce childhood obesity. Regarding the associations of CEBQs and dietary intake, Food Responsiveness (FR) was positively associated with total calorie (TC) and Enjoyment of Food (EF) was also positively correlated with TC and fat intakes. High consumption of energy-dense foods stimulates the release of dopamine and serotonin, creating a cycle of rewards that encourages the excessive consumption of these foods [41]. Flavoured foods not only disrupt satiety responses but also have the potential to activate the brain’s reward systems [42], thereby reinforcing increased food intake even in the absence of physiological energy needs. Consequently, elevated FR and EF scores may contribute to the development of obesity in children. DD was linked with higher cholesterol intake, which may be attributed to the consumption of high-energy palatable beverages. These stimulate the brain’s reward system, promoting increased intake. Additionally, beverages require less oral processing and stimulate lower GLP-1 and Peptide YY (PYY) secretion, leading to faster gastric emptying and intestinal transit compared to solid or protein-rich foods [43]. Our study found that increased GLP-1 hormone at final visit was associated with lower EOE score. GLP-1 plays a key role in appetite regulation, primarily through its receptors in the hypothalamus. Administration of GLP-1 hormone or GLP-1 receptor agonists (GLP-1RAs) in human has been shown to reduce food intake and appetite while enhancing feelings of fullness and satiety [44]. Long-term study revealed that GLP-1RAs decreased cravings for high-calorie and sweet foods, reduced overall desire to eat, and contributed to significant weight loss after two years [45]. These indicate the potential of GLP-1 and GLP-1RAs in regulating appetite and related eating behaviours, thereby supporting weight management.

Furthermore, we reported significant correlations between subjective eating behaviours and gut microbiota. The associations at the genus level showed that EOE was negatively associated with *Agathobacter* and *Oscillibacter*. *Agathobacter*, a butyrate-producing genus, has the ability to break down non-digestible carbohydrates in the human gastrointestinal tract [46] and was found to increase following prebiotic supplementation in children with obesity [36]. This genus has also been identified in adults with obesity who achieved weight control after 12 weeks of high-fiber rye administration [46] and in those receiving oatmeal [47]. *Oscillibacter*, a valerate-producing bacterium, generates short chain fatty acids (SCFAs) metabolites that inhibit histone deacetylase, contributing to disease management. A previous study found its relative abundance to be positively associated with high fiber intake [48]. EOE was associated with the consumption of hyperpalatable, calorie-dense foods. Therefore, negative emotional stress, which drives excessive food intake, may be linked to a reduction in the abundance of these bacteria. We found that food approach subscales were negatively correlated with *UBA1819*, which has been linked to beneficial effects on metabolic health. A previous study reported an increased abundance of *UBA1819* following prebiotic treatment [49]. Similarly, milled flaxseed feeding has been shown to enhance the relative abundance of *UBA1819*, potentially contributing to its anti-inflammatory effects [50]. These findings indicate the role of *UBA1819* in improving metabolic outcomes and its inverse association with food approach behaviours. In addition, Satiety Responsiveness was negatively associated with *Lachnospiraceae_NK4A136*. Recent study has shown that individuals living with obesity exhibit a higher abundance of *Lachnospiraceae*, which has been associated with type 2 diabetes and obesity [51]. *Lachnospiraceae* can be enriched by a high saturated fat and low fiber diet [52]. This highlights the role of dietary patterns and eating habits in shaping gut microbial composition and diversity. In turn, gut microbiota appears to be associated with eating behaviours, reflecting a bidirectional relationship between diet, gut flora, and food-related habits.

The strengths of the present study include its status as the largest randomized, double-blinded, placebo-controlled trial, designed to minimize bias and ensure high compliance with the intervention, which demonstrated a significant effect of inulin supplementation on eating behaviours. Another notable strength is that this study was the first to explore associations between eating behaviour assessments, dietary intake and patterns, and gut microbiota in children with obesity, offering insights into the bidirectional relationship between eating behaviours, diet, and gut microbial composition. However, the study has some limitations. Firstly, we did not assess certain gut hormones that regulate appetite, such as ghrelin, adiponectin, and leptin, which may provide a more comprehensive understanding of the mechanisms by which inulin supplementation influences eating behaviours. Secondly, although the analyses yielded statistically significant associations, the corresponding Spearman’s rho coefficients indicate that these correlations are relatively weak. Therefore, careful consideration is needed in interpreting these findings, as the observed relationships might not imply causation. Thirdly, while eating behaviour questionnaires offer valuable insights into eating habits. They should be used in conjunction with other objective measurements to obtain a comprehensive understanding of an individual’s eating patterns and their relationship to clinical parameters. Fourthly, this was a three-arm randomized controlled trial including intervention and placebo groups which were blinded to group allocation to preserve the double-blind design. However, the dietary fiber advice group did not receive a placebo or supplement; therefore, participants in this arm were necessarily aware of their allocation. Lastly, despite all participants in this research received the same dietary guidance, nevertheless, we acknowledge that providing age-tailored recommendations might enhance adherence and outcomes, and this may be considered in future studies.

## Conclusions

Our study highlights that inulin supplementation might be a potential intervention for managing childhood obesity by modulating appetite and improving eating behaviours. Additionally, this is the first study to identify the relationships of dietary intakes and eating habits with gut microbiota diversity in this population. Further research is needed to explore how prebiotics influence gut hormones underlying eating behaviours, which could inform more effective interventions for childhood obesity.

## Supplementary Information

Below is the link to the electronic supplementary material.

Supplementary Material 1

Supplementary Material 2

## Data Availability

The datasets used and/or analysed during the current study are available from the corresponding author on reasonable request.

## Abbreviations

ANOVA: Analysis of Variance
BMI: Body mass index
CEBQs: Children’s eating behaviour questionnaires
DD: Desire to drink
DF/Kcal: Dietary fiber intake per 1,000 kcal
DNA: Deoxyribonucleic acid
EF: Enjoyment of food
EOE: Emotional overeating
EUE: Emotional undereating
FATi: Fat intakes
FF: Food fussiness
FR: Food responsiveness
GLP-1: Glucagon like peptide-1
GLP-1RAs: Glucagon like peptide-1 receptor agonists
ITFs: Inulin-type fructans
LDA: Linear discriminant analysis
LEfSe: Linear Discriminant Analysis Effect Size
pCHO: Proportion of carbohydrate intake
PYY: Peptide YY
rRNA: Ribosomal ribonucleic acid
SCFAs: Short chain fatty acids
SDs: Standard deviations
SE: Slowness in eating
SR: Satiety responsiveness
TC: Total calorie
WHO: World Health Organization

## References

[1] World, Health O. Obesity and overweight Geneva, Switzerland 2021 [updated 9 June 2021]. https://www.who.int/news-room/fact-sheets/detail/obesity-and-overweight. Accessed 24 April 2024.

[2] Pongcharoen T, Rojroongwasinkul N, Tuntipopipat S, Winichagoon P, Vongvimetee N, Phanyotha T, et al. South East Asian nutrition surveys II (SEANUTS II) thailand: triple burden of malnutrition among Thai children aged 6 months to 12 years. Public Health Nutr. 2024;27(1):e152. 38250788 10.1017/S1368980024000053 PMC11617422

[3] Hampl SE, Hassink SG, Skinner AC, Armstrong SC, Barlow SE, Bolling CF, et al. Clinical practice guideline for the evaluation and treatment of children and adolescents with obesity. Pediatrics. 2023;151(2):e2022060640. 36622115 10.1542/peds.2022-060640

[4] Galler A, Thönnes A, Joas J, Joisten C, Körner A, Reinehr T, et al. Clinical characteristics and outcomes of children, adolescents and young adults with overweight or obesity and mental health disorders. Int J Obes. 2024;48(3):423–32. 10.1038/s41366-023-01449-4 PMC10896720 38195831

[5] Burgess B, Faith MS. Satiety responsiveness and eating rate in childhood: development, plasticity, and the family footprint. In: Julie C, Lumeng JO, Fisher, editors. Pediatric food preferences and eating behaviors. Cambridge, MA: Elsevier; 2018. pp. 93–106.

[6] Webber L, Hill C, Saxton J, Van Jaarsveld CH, Wardle J. Eating behaviour and weight in children. Int J Obes (Lond). 2009;33(1):21–8. 19002146 10.1038/ijo.2008.219 PMC2817450

[7] Wardle J, Guthrie CA, Sanderson S, Rapoport L. Development of the children’s eating behaviour questionnaire. J Child Psychol Psychiatry. 2001;42(7):963–70. 11693591 10.1111/1469-7610.00792

[8] Santos JL, Ho-Urriola JA, González A, Smalley SV, Domínguez-Vásquez P, Cataldo R, et al. Association between eating behavior scores and obesity in Chilean children. Nutr J. 2011;10:108. 21985269 10.1186/1475-2891-10-108 PMC3213088

[9] Malczyk Ż, Pasztak-Opiłka A, Zachurzok A. Different eating habits are observed in overweight and obese children than in normal-weight peers. Child (Basel). 2024;11(7):834. 10.3390/children11070834 PMC11276219 39062283

[10] Favieri F, Marini A, Casagrande M. Emotional regulation and overeating behaviors in children and adolescents: a systematic review. Behav Sci (Basel). 2021;11(1):11. 33477932 10.3390/bs11010011 PMC7833366

[11] Hughes RL, Alvarado DA, Swanson KS, Holscher HD. The prebiotic potential of inulin-type fructans: a systematic review. Adv Nutr. 2022;13(2):492–529. 34555168 10.1093/advances/nmab119 PMC8970830

[12] Visuthranukul C, Chamni S, Kwanbunbumpen T, Saengpanit P, Chongpison Y, Tepaamorndech S, et al. Effects of inulin supplementation on body composition and metabolic outcomes in children with obesity. Sci Rep. 2022;12(1):13014. 35906473 10.1038/s41598-022-17220-0 PMC9338247

[13] Visuthranukul C, Leelahavanichkul A, Tepaamorndech S, Chamni S, Mekangkul E, Chomtho S. Inulin supplementation exhibits increased muscle mass via gut-muscle axis in children with obesity: double evidence from clinical and in vitro studies. Sci Rep. 2024;14(1):11181. 38755201 10.1038/s41598-024-61781-1 PMC11099025

[14] Koh A, De Vadder F, Kovatcheva-Datchary P, Bäckhed F. From dietary fiber to host physiology: short-chain fatty acids as key bacterial metabolites. Cell. 2016;165(6):1332–45. 27259147 10.1016/j.cell.2016.05.041

[15] Parnell JA, Reimer RA. Prebiotic fiber modulation of the gut microbiota improves risk factors for obesity and the metabolic syndrome. Gut Microbes. 2012;3(1):29–34. 22555633 10.4161/gmic.19246 PMC3827018

[16] Reimer RA, Theis S, Zanzer YC. The effects of chicory inulin-type fructans supplementation on weight management outcomes: systematic review, meta-analysis, and meta-regression of randomized controlled trials. Am J Clin Nutr. 2024;120(5):1245–58. 39313030 10.1016/j.ajcnut.2024.09.019 PMC11600113

[17] Reimer RA, Willis HJ, Tunnicliffe JM, Park H, Madsen KL, Soto-Vaca A. Inulin-type fructans and whey protein both modulate appetite but only fructans alter gut microbiota in adults with overweight/obesity: a randomized controlled trial. Mol Nutr Food Res. 2017;61(11). 10.1002/mnfr.201700484 10.1002/mnfr.201700484 28730743

[18] Daud NM, Ismail NA, Thomas EL, Fitzpatrick JA, Bell JD, Swann JR, et al. The impact of oligofructose on stimulation of gut hormones, appetite regulation and adiposity. Obes (Silver Spring). 2014;22(6):1430–8. 10.1002/oby.20754 24715424

[19] Pol K, de Graaf C, Meyer D, Mars M. The efficacy of daily snack replacement with oligofructose-enriched granola bars in overweight and obese adults: a 12-week randomised controlled trial. Br J Nutr. 2018;119(9):1076–86. 29490721 10.1017/S0007114518000211

[20] Hume MP, Nicolucci AC, Reimer RA. Prebiotic supplementation improves appetite control in children with overweight and obesity: a randomized controlled trial. Am J Clin Nutr. 2017;105(4):790–9. 28228425 10.3945/ajcn.116.140947

[21] Ross AC, Caballero B, Cousins RJ, Tucker KL. TR. Z. Modern nutrition in health and disease. 11 ed. Philadelphia, PA: Lippincott Williams & Wilkins; 2012.

[22] Bureau of Nutrition, Department of Health, Ministry of Public Health. Food Composition Table of Thai Foods 2018. https://nutrition2.anamai.moph.go.th/th/thai-food-composition-table. Accessed 22 November 2024.

[23] Banjong O, Wanijjaul C. K. Peemanee. Application Manual:INMUCAL-Nutrients V.3. Institute of nutrition. Mahidol University; 2016.

[24] Monika BA, Elaine B. O. Adelheid, d. Mercedes, onis. WHO anthroplus for personal computers manual: software for assessing growth of the world’s children and adolescents. Geneva, Switzerland: Department of Nutrition for Health and Development, World Health Organization; 2009.

[25] Panichsillaphakit E, Chongpison Y, Saengpanit P, Kwanbunbumpen T, Uaariyapanichkul J, Chomtho S, et al. Children’s eating behavior questionnaire correlated with body compositions of Thai children and adolescents with obesity: a pilot study. J Nutr Metab. 2021;2021:6496134. 33510908 10.1155/2021/6496134 PMC7822704

[26] Sirirassamee T, Hunchangsith P. Children’s eating behavior questionnaire: factorial validation and differences in sex and educational level in Thai school-age children. Southeast Asian J Trop Med Public Health. 2016;47(6):1325–34. 29634198

[27] Visuthranukul C, Sriswasdi S, Tepaamorndech S, Joyjinda Y, Saengpanit P, Kwanbunbumpen T, et al. Association of human intestinal microbiota with lifestyle activity, adiposity, and metabolic profiles in Thai children with obesity. J Nutr Metab. 2022;2022:3029582. 35637874 10.1155/2022/3029582 PMC9146442

[28] Bolyen E, Rideout JR, Dillon MR, Bokulich NA, Abnet CC, Al-Ghalith GA, et al. Reproducible, interactive, scalable and extensible microbiome data science using QIIME 2. Nat Biotechnol. 2019;37(8):852–7. 31341288 10.1038/s41587-019-0209-9 PMC7015180

[29] Babraham IB. Bioinformatics. 2021. https://www.bioinformatics.babraham.ac.uk/projects/fastqc/. Accessed 15 October 2024.

[30] Callahan BJ, McMurdie PJ, Rosen MJ, Han AW, Johnson AJ, Holmes SP. DADA2: high-resolution sample inference from illumina amplicon data. Nat Methods. 2016;13(7):581–3. 27214047 10.1038/nmeth.3869 PMC4927377

[31] Bokulich NA, Kaehler BD, Rideout JR, Dillon M, Bolyen E, Knight R, et al. Optimizing taxonomic classification of marker-gene amplicon sequences with QIIME 2’s q2-feature-classifier plugin. Microbiome. 2018;6(1):90. 29773078 10.1186/s40168-018-0470-z PMC5956843

[32] Robeson MS 2nd, O’Rourke DR, Kaehler BD, Ziemski M, Dillon MR, Foster JT, et al. RESCRIPt: reproducible sequence taxonomy reference database management. PLoS Comput Biol. 2021;17(11):e1009581. 34748542 10.1371/journal.pcbi.1009581 PMC8601625

[33] Segata N, Huttenhower C. Toward an efficient method of identifying core genes for evolutionary and functional microbial phylogenies. PLoS ONE. 2011;6(9):e24704. 21931822 10.1371/journal.pone.0024704 PMC3171473

[34] Lin X, Li H, Obesity. Epidemiology, pathophysiology, and therapeutics. Front Endocrinol (Lausanne). 2021;12:706978. 34552557 10.3389/fendo.2021.706978 PMC8450866

[35] Jackson PPJ, Wijeyesekera A, Williams CM, Theis S, van Harsselaar J, Rastall RA. Inulin-type fructans and 2’fucosyllactose alter both microbial composition and appear to alleviate stress-induced mood state in a working population compared to placebo (maltodextrin): the EFFICAD trial, a randomized, controlled trial. Am J Clin Nutr. 2023;118(5):938–55. 37657523 10.1016/j.ajcnut.2023.08.016 PMC10636234

[36] Visuthranukul C, Sriswasdi S, Tepaamorndech S, Chamni S, Leelahavanichkul A, Joyjinda Y, et al. Enhancing gut microbiota and microbial function with inulin supplementation in children with obesity. Int J Obes (Lond). 2024;48(12):1696–704. 39033197 10.1038/s41366-024-01590-8 PMC11584386

[37] Huang F, Wu X. Brain neurotransmitter modulation by gut microbiota in anxiety and depression. Front Cell Dev Biol. 2021;9:649103. 33777957 10.3389/fcell.2021.649103 PMC7991717

[38] Dovey TM, Staples PA, Gibson EL, Halford JC. Food neophobia and ‘picky/fussy’ eating in children: a review. Appetite. 2008;50(2–3):181–93. 17997196 10.1016/j.appet.2007.09.009

[39] Dubois L, Farmer A, Girard M, Peterson K, Tatone-Tokuda F. Problem eating behaviors related to social factors and body weight in preschool children: a longitudinal study. Int J Behav Nutr Phys Act. 2007;4:9. 17408478 10.1186/1479-5868-4-9 PMC1855064

[40] Hayes JF, Altman M, Kolko RP, Balantekin KN, Holland JC, Stein RI, et al. Decreasing food fussiness in children with obesity leads to greater weight loss in family-based treatment. Obes (Silver Spring). 2016;24(10):2158–63. 10.1002/oby.21622 PMC5039088 27601189

[41] Cîmpeanu RC, Caragea EM, Mustață LM, Forțofoiu D, Dragne IG, Alexa RE, et al. The involvement of serotonin in the obesity pathway-a last decade systematic review of the literature. Int J Mol Sci. 2025;26(7):3081. 40243845 10.3390/ijms26073081 PMC11988484

[42] Freitas A, Albuquerque G, Silva C, Oliveira A. Appetite-related eating behaviours: an overview of assessment methods, determinants and effects on children’s weight. Ann Nutr Metab. 2018;73(1):19–29. 29843129 10.1159/000489824

[43] Costa D, Warkentin S, Oliveira A. Sugar-sweetened beverages, effects on appetite and public health strategies to reduce the consumption among children: a review. Porto Biomed J. 2022;7(1):e172. 35146179 10.1097/j.pbj.0000000000000172 PMC8824388

[44] Bodnaruc AM, Prud’homme D, Blanchet R, Giroux I. Nutritional modulation of endogenous glucagon-like peptide-1 secretion: a review. Nutr Metab (Lond). 2016;13:92. 27990172 10.1186/s12986-016-0153-3 PMC5148911

[45] Wharton S, Batterham RL, Bhatta M, Buscemi S, Christensen LN, Frias JP, et al. Two-year effect of semaglutide 2.4 mg on control of eating in adults with overweight/obesity: STEP 5. Obes (Silver Spring). 2023;31(3):703–15. 10.1002/oby.23673 36655300

[46] Iversen KN, Dicksved J, Zoki C, Fristedt R, Pelve EA, Langton M, et al. The effects of high fiber rye, compared to refined wheat, on gut microbiota composition, plasma short chain fatty acids, and implications for weight loss and metabolic risk factors (the Ryeweight study). Nutrients. 2022;14(8):1669. 35458231 10.3390/nu14081669 PMC9032876

[47] Xu D, Pan D, Liu H, Yang C, Yang X, Wang X, et al. Improvement in cardiometabolic risk markers following an oatmeal diet is associated with gut microbiota in mildly hypercholesterolemic individuals. Food Res Int. 2022;160:111701. 36076452 10.1016/j.foodres.2022.111701

[48] Rosés C, Cuevas-Sierra A, Quintana S, Riezu-Boj JI, Martínez JA, Milagro FI, et al. Gut microbiota bacterial species associated with mediterranean diet-related food groups in a Northern Spanish population. Nutrients. 2021;13(2):636. 33669303 10.3390/nu13020636 PMC7920039

[49] Obermüller B, Singer G, Kienesberger B, Klymiuk I, Sperl D, Stadlbauer V, et al. The effects of prebiotic supplementation with OMNi-LOGiC(^®^) FIBRE on fecal microbiome, fecal volatile organic compounds, and gut permeability in murine neuroblastoma-induced tumor-associated cachexia. Nutrients. 2020;12(7):2029. 32650568 10.3390/nu12072029 PMC7400931

[50] Hui Xia XS, Beijia Zhou J, Sui C, Yang H, Liu L, Yang S, Wang G, Sun. Milled flaxseed-added diets ameliorated hepatic inflammation by reducing gene expression of TLR4/NF-κB pathway and altered gut microbiota in STZ-induced type 1 diabetic mice. Food Sci Hum Well. 2022;11(1):32–40.

[51] Ottosson F, Brunkwall L, Ericson U, Nilsson PM, Almgren P, Fernandez C, et al. Connection between BMI-related plasma metabolite profile and gut microbiota. J Clin Endocrinol Metab. 2018;103(4):1491–501. 29409054 10.1210/jc.2017-02114

[52] Vacca M, Celano G, Calabrese FM, Portincasa P, Gobbetti M, De Angelis M. The controversial role of human gut lachnospiraceae. Microorganisms. 2020;8(4):573. 32326636 10.3390/microorganisms8040573 PMC7232163

