# Supplementary material for: The effects of inulin supplementation on eating behaviours in children and adolescents with obesity: a randomized double-blinded placebo-controlled study
Source: Nutr Metab (Lond). 2025 Aug 12;22:97. doi: 10.1186/s12986-025-00995-0 (PMC12341223; doi:10.1186/s12986-025-00995-0)
Supplement: Supplementary file 1 — Supplementary Material 1 [file 12986_2025_995_MOESM1_ESM.docx]

**
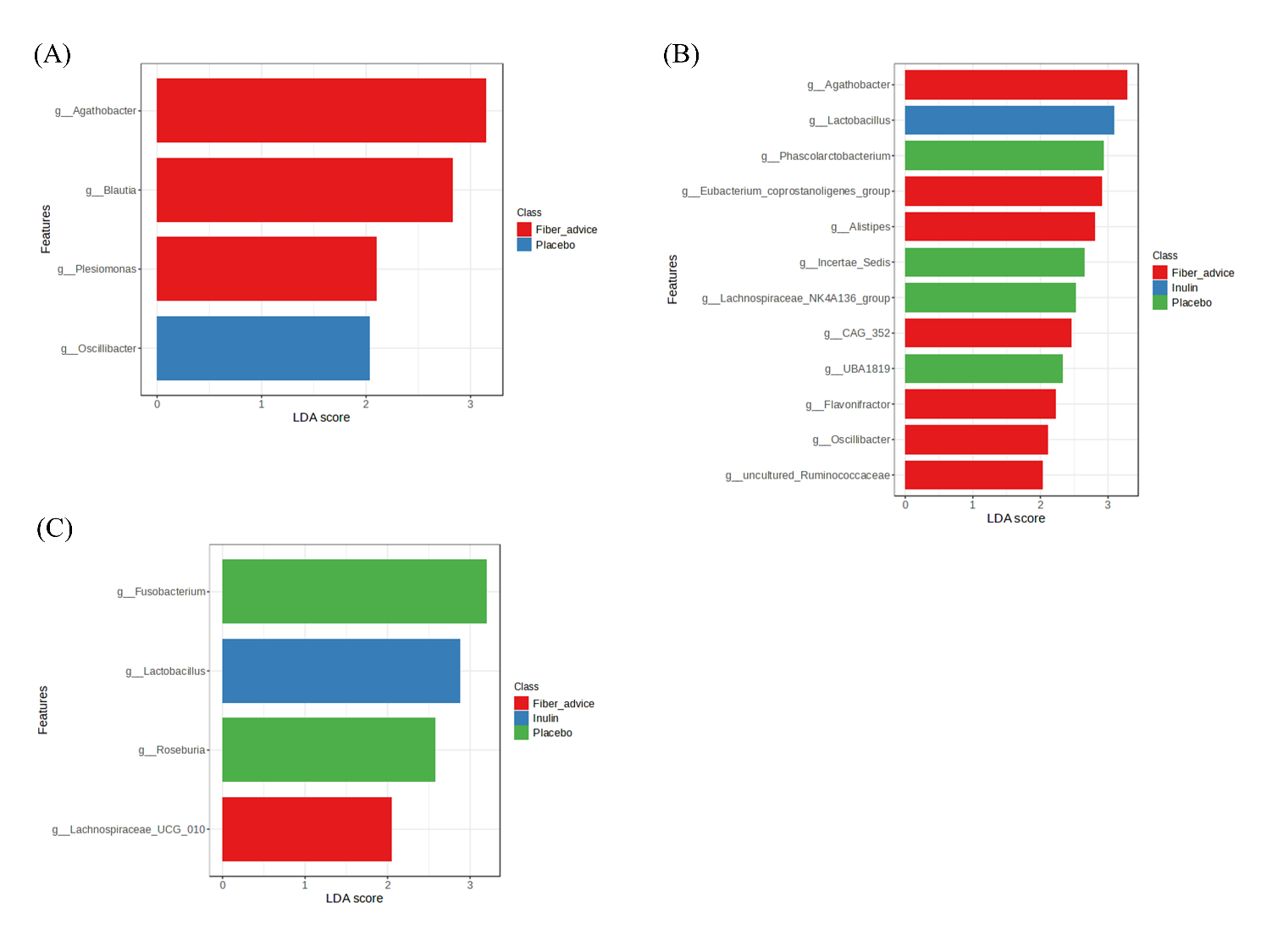
**

**Supplementary Fig 1.** LEfSe analysis for identifying the bacteria enrichment among groups (cut-off LDA score ≥ 2.0, p-value <0.05). (**A**) At baseline, (**B**) At month 3 and **(C)** At month 6

*Abbreviations: LEfSe*, Linear Discriminant Analysis Effect Size; *LDA*, linear discriminant analysis
