# Supplementary material for: The effects of inulin supplementation on eating behaviours in children and adolescents with obesity: a randomized double-blinded placebo-controlled study
Source: Nutr Metab (Lond). 2025 Aug 12;22:97. doi: 10.1186/s12986-025-00995-0 (PMC12341223; doi:10.1186/s12986-025-00995-0)

Study flow diagram

Manuscript entitles: The effects of inulin supplementation on eating behaviours in children and adolescents with obesity: A randomized double-blinded placebo-controlled study

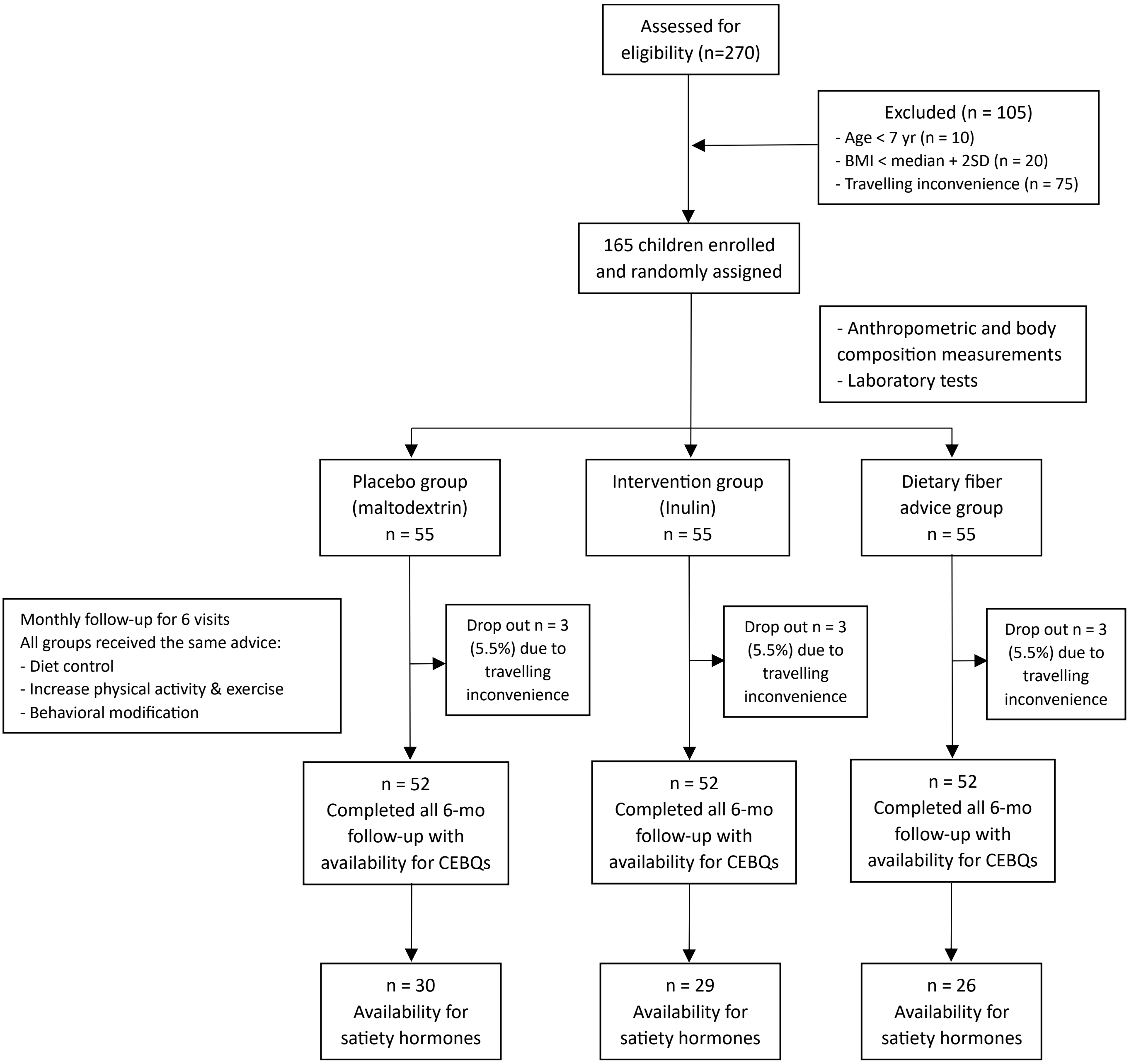

Supplement: Supplementary file 2 — Supplementary Material 2 [file 12986_2025_995_MOESM2_ESM.pdf]
